# Elucidating Drought Stress Tolerance in European Oaks Through Cross-Species Transcriptomics

**DOI:** 10.1534/g3.119.400456

**Published:** 2019-08-08

**Authors:** Silvia Madritsch, Elisabeth Wischnitzki, Peter Kotrade, Ahmed Ashoub, Agnes Burg, Silvia Fluch, Wolfgang Brüggemann, Eva M. Sehr

**Affiliations:** * Center for Health & Bioresources, AIT Austrian Institute of Technology GmbH, 3430 Tulln, Austria,; † Center for Integrative Bioinformatics Vienna, Max Perutz Labs, University of Vienna, Medical University of Vienna, 1030 Vienna, Austria,; ‡ Department of Ecology, Evolution and Diversity, Goethe University Frankfurt, 60438 Frankfurt, Germany, and; § Senckenberg Biodiversity and Climate Research Centre (BIK-F), 60325 Frankfurt, Germany

**Keywords:** Drought stress response, *Quercus robur*, *Quercus pubescens*, *Quercus ilex*, antioxidant capacity

## Abstract

The impact of climate change that comes with a dramatic increase of long periods of extreme summer drought associated with heat is a fundamental challenge for European forests. As a result, forests are expected to shift their distribution patterns toward north-east, which may lead to a dramatic loss in value of European forest land. Consequently, unraveling key processes that underlie drought stress tolerance is not only of great scientific but also of utmost economic importance for forests to withstand future heat and drought wave scenarios. To reveal drought stress-related molecular patterns we applied cross-species comparative transcriptomics of three major European oak species: the less tolerant deciduous pedunculate oak (*Quercus robur*), the deciduous but quite tolerant pubescent oak (*Q. pubescens*), and the very tolerant evergreen holm oak (*Q. ilex*). We found 415, 79, and 222 differentially expressed genes during drought stress in *Q. robur*, *Q. pubescens*, and *Q. ilex*, respectively, indicating species-specific response mechanisms. Further, by comparative orthologous gene family analysis, 517 orthologous genes could be characterized that may play an important role in drought stress adaptation on the genus level. New regulatory candidate pathways and genes in the context of drought stress response were identified, highlighting the importance of the antioxidant capacity, the mitochondrial respiration machinery, the lignification of the water transport system, and the suppression of drought-induced senescence – providing a valuable knowledge base that could be integrated in breeding programs in the face of climate change.

Climate change represents a fundamental challenge for European forests. Apart from the expected increase in average temperatures, a key stressor of European forests will be the increasing probability of extreme summer drought associated with heat, like in the summer of 2003. Especially forests dominated by relatively drought-sensitive coniferous and broadleaf species (*i.e.*, Norway spruce, Scots pine, European beech), but also mixed oak forests at the dry fringes of the Central European beech belt and in the Mediterranean countries will be subjected to increasing frequencies of heat waves and changes in spatial and temporal precipitation patterns. As a consequence, a north-east shift of their distribution pattern has to be expected (Liu *et al.* 2018) and will be associated with a dramatic loss in value of European forest land, probably reaching several hundred billion EUR in the period to 2100 (Hickler *et al.* 2012; Hanewinkel *et al.* 2013). Increased drought stress (DS) tolerance of forest trees will be a key feature to maintain functional forest cover in climate-threatened regions. Unraveling key processes that underlie DS tolerance is not only of great scientific but also of utmost economic and ecologic importance. Especially the development of molecular markers that are linked to DS tolerance offer the potential to accelerate breeding programs or assisted migration projects through marker assisted selection (Butcher and Southerton 2007).

Several omics analyses (transcriptomic, proteomic, and metabolomic) have already been performed to identify drought-related patterns as well as their associated signaling and regulatory pathways in plants (Joshi *et al.* 2016), including various tree species (Harfouche *et al.* 2014). However, despite the high economic relevance of oaks as forestry species in Europe, molecular mechanisms of DS tolerance have explicitly been investigated so far only by a limited number of studies: in *Quercus robur*, transcriptomic and proteomic responses to long-term drought exposure elucidated the involvement of genes with protective functions, such as chaperones and dehydrins (Sunderlíková *et al.* 2009; Spieß *et al.* 2012) and highlighted the degradation of RuBisCO (Sergeant *et al.* 2011). Porth *et al.* (2005) assessed differences in DS responses in *Q. robur* and *Q. petraea* by analyzing a set of known osmotic-stress-induced transcripts. In addition, the high natural variation in DS related genes of *Q. robur* in contrast to *Q. petraea* provided first evidences of natural selection in the frame of drought adaptation processes (Homolka *et al.* 2012, 2013). This concept of local adaptation to changing environments is supported by a study of three European white oak species (*Q. robur*, *Q. petraea* and *Q. pubescens*) by Rellstab *et al.* (2016). In *Q. suber*, an activation of abscisic acid (ABA) responsive genes and ABA-dependent signaling was characterized in the root transcriptome in response to drought (Magalhães *et al.* 2015), and an increase of cytosine methylation was recognized in heat-treated leaves (Correia *et al.* 2013). A detailed analysis of the transcription factor QsMYB1 highlighted its role in the response to heat and drought stress in this species (Almeida *et al.* 2013). Finally, various studies were performed to elucidate the response to DS of *Q. ilex*, reporting the involvement of enzymes of carbohydrate and protein metabolism (Jorge *et al.* 2006), an increase in stress response proteins such as those involved in detoxifying reactive oxygen species (Echevarría-Zomeño *et al.* 2009), an increase of proline and γ-Aminobutyric acid (GABA) in severely drought stressed seedlings (Rodríguez-Calcerrada *et al.* 2017), increased concentrations of sugars and polyphenolic compounds in leaves (Rivas-Ubach *et al.* 2014), and a down-regulation of glycolysis and stimulation of ATP synthesis in roots and seedlings subjected to water limitation (Valero-Galván *et al.* 2013; Simova-Stoilova *et al.* 2015). Despite this seemingly long list, a comprehensive species-spanning comparative analysis of DS response has not been performed so far.

Although oaks are generally considered to be DS tolerant, the level of tolerance differs considerably among oak species, reflecting their broad adaptation potential to environments with differing soil moisture regimes. In the current study we took advantage of the present DS tolerance gradient from the less tolerant pedunculate oak (*Q. robur*) via the quite tolerant pubescent oak (*Q. pubescens*) to the very tolerant holm oak (*Q. ilex*). In addition to their contrasting geographical ranges (pedunculate oak is a temperate–boreal species, holm oak a Mediterranean species and pubescent oak has an intermediate distribution), the three species are characterized by a different leaf habit, whereby pedunculate oak and pubescent oak are deciduous while holm oak is evergreen. All these differences were considered to unveil new regulatory candidate pathways and genes in the context of DS response by means of comparative transcriptomics, from the species up to the genus level.

## Materials and Methods

### Plant material and experimental site

For the purpose of comparative transcriptomics, three valuable European oak species with differing DS tolerance were chosen: *Q. robur* L. (pedunculate oak), a common deciduous broad leaved species showing a wide distribution range across Europe (Eaton *et al.* 2016), *Q. pubescens* Willd., the downy or pubescent oak, a more thermophilous (semi-) deciduous oak species which occupies almost all of Southern Europe, ranging into Western and Central Europe (Pasta *et al.* 2016), and the evergreen holm oak, *Q. ilex* L., a sclerophyllous species well adapted to drought occurring in the Central-Western Mediterranean basin (de Rigo and Caudullo 2016). Three to five 9-year-old plants of *Q. robur* (provenance: Mitteldeutsches Tief- und Hügelland D-81705), *Q. pubescens* (provenance: Languedoc F-QPU701), and *Q. ilex* subsp. *ilex* (provenance: Languedoc F-QIL 702) were grown outdoors in two basins (6*4.5*2 m^3^ each) for two years for root development from 2012 onwards in central Germany (50°10’08.9”N 8°37’48.6”E). The basins were filled with silty loam and covered with a semi cylindrical foil tunnel to allow irrigation control. Temperature, RH and solar radiation were captured by a weather station beside the basins. Monitoring of water tables in the basins was enabled by floaters within drainage pipes on the ground of the basins and kept at -1.8 m throughout the root development phase. Further details can be found in Früchtenicht *et al.* (2018b) and Kotrade *et al.* (2019).

### Drought stress treatment and monitoring

One basin was chosen for the drought stress (DS) treatment, the other one as control (CO). In 2014, irrigation of the DS basin was stopped on DOY (day of year) 170 for 124 days while the CO basin was kept on a constant water table. The DS level in the plants was monitored by measuring predawn water potential (Ψ_PD_) with a scholander pressure bomb (Skye Instruments Ltd, UK) and the performance index (PIabs) with a Pocket PEA (Hansatech Instruments Ltd, UK). More detailed information on the experimental design, the environmental and physiological data were recently published in Früchtenicht *et al.* (2018b) and Kotrade *et al.* (2019).

### Sampling and RNA extraction

Sampling was done on DOY 287 (*Q. robur*, *Q. pubescens*) and 288 (*Q. ilex*) in 2014 when Ψ_PD_ values showed significant differences between CO and DS groups in all species (for details see Kotrade *et al.* 2019). Leaves were taken from three trees per treatment and species, except for *Q. robur*, where only two trees of the CO group were sampled, since one individual perished. Per tree, three to five fully developed leaves of upper branches in southern orientation were sampled. For the evergreen species *Q. ilex*, only recent leaves developed in 2014 were collected. Leaves were immediately shock-frozen in liquid nitrogen and kept at -80° until RNA extraction. A modified CTAB-based RNA extraction protocol was used to extract RNA from leaves pooled for each tree (see Kotrade *et al.* 2019).

### Library preparation and sequencing

Extracted total RNA was sent to VBCF NGS Unit (www.viennabiocenter.org/facilities) for library preparation and Illumina RNA sequencing. An overview of the samples sent for sequencing is given in Table S1. Shortly, after RNA quality control (Agilent 2100 Bioanalyzer), RNA library preparation of the 17 samples was done using the polyA enrichment strategy according to the manufacturer’s protocol (NEB chemistry), pooled and sequenced together on three lanes (technical replicates). Sequencing of the first two lanes was done on a HiSeq 2500 as 150 bp PE reads in rapid mode, the third lane was sequenced as 125 PE reads.

### Quality control and pre-processing of sequencing data

All sequence reads were cleaned in order to guarantee high quality (HQ) data by removing remaining reads derived from the phiX genome (NCBI accession NC_001422) (Sanger *et al.* 1977), ambiguous reads, low quality regions (Q30), and short sequences (< 50 bp) from the datasets using bowtie2 (Langmead and Salzberg 2012), SAMtools (Li *et al.* 2009), and BEDtools (Quinlan and Hall 2010). Additionally, all HQ read pairs were tested whether their ends overlap using Flash with a minimal overlap of 10 bp and a maximal overlap of 250 bp (Magoč and Salzberg 2011).

### De novo assembly

By combining all datasets (CO and DS) of each species, a *de novo* assembly was performed using Trinity (Grabherr *et al.* 2011; Haas *et al.* 2013) with default values and a minimal contig length of 100 bp to establish a species-specific leaf reference transcriptome. The resulting contigs were evaluated by mapping the pre-processed reads used for the assembly back to the assembled contig sequences using bowtie2 with default settings (Langmead and Salzberg 2012) and only those contigs consisting of at least five reads were retained.

### Differential gene expression

Species-specific transcript abundance estimation was performed using RSEM (Li and Dewey 2011). RSEM performs abundance calculations on isoforms and genes. The gene expression level, that were used for further analysis is the sum of isoform expression levels. Additionally, RSEM outputs two files, one with the expected counts, that were used for DEG analysis with edgeR and one with TPM values (normalized by length) that were used comparing expression across the species (see below). CPM (counts per million mapped reads) values for each sample were calculated from expected counts using edgeR’s cpm function. For annotation and differential gene expression only those genes were included that have a CPM greater than one in at least two samples. After filtering, further expression analysis was performed using RSEM expected counts and edgeR exactTest (Robinson *et al.* 2010; McCarthy *et al.* 2012) as well as p-value correction for multiple testing (FDR) with the Benjamini and Hochberg (BH) procedure. Significant genes were considered with |logFC| > 1 and a FDR < 0.05.

### Functional annotation and classification

The *de novo* assembly filtered for low expressed genes were functionally annotated and classified as follows: Open reading frames (ORFs) were predicted using TransDecoder with a minimal length of 100 amino acids (Magoč and Salzberg 2011). Similarity searches were performed using BLASTX and BLASTP against the UniProt (Swiss-Prot) and against NCBI non-redundant Viridiplantae databases. Gene Ontology (GO) and KEGG pathways were assigned to the annotated BLASTX results using Trinotate (McCarthy *et al.* 2012). Similarities were considered significant with E-values ≤ 1e-5. The presence of known functional protein domains was performed using HMMER (Eddy 1998) and the Pfam protein domain database.

### GO enrichment analysis

Functional over-representation analysis of significant differentially expressed genes was performed using the R-package TopGO with the Fisher’s exact test (Alexa *et al.* 2006). Gene Ontology (GO) categories are declared as significantly over-represented with a p-value level of < 0.01. GO enrichment visualization was performed using REVIGO (Supek *et al.* 2011).

### Gene expression among species

The three species-specific assembled transcriptomes (including low expressed transcripts with CPM < 1) were compared to each other to identify orthologous genes in each of the other species transcriptomes using ORTHOfinder with default parameters (Emms and Kelly 2015). Only orthogroups that contain at least one gene of each species were considered for further analysis. Differential gene expression comparison among *Quercus* species was performed with exploratory data analysis methods. To compute the species-wise gene expression level for each orthologous group, normalized gene expression levels (TPM) of all genes were summed up per species. A pairwise comparison (*Q. robur - Q. pubescens*, *Q. robur - Q. ilex and Q. pubescens - Q. ilex*) has been performed with the workflow of the R package crossr (Mantegazza 2018) to find orthogroups that behave differently under DS condition between the species. Crossr fits a linear model and performs ANOVA (design formula: ∼species + treatment + species:treatment). The 200 orthologous groups with highest F-values of each pair from the ANOVA test were selected and used for further visualization.

### Data availability

The datasets generated and analyzed in the current study are available in the NCBI SRA (Sequence Read Archive) and TSA (Transcriptome Shotgun Assembly), BioProject: PRJNA450334 (https://www.ncbi.nlm.nih.gov/bioproject/PRJNA450334). Only transcripts greater 199 nt were uploaded. Supplemental material available at FigShare: https://doi.org/10.25387/g3.8288915.

## Results

### Physiological status of drought stressed plants

The three trees species were grown together in loamy soil in 2 m deep basins in a competition experiment setup, thus experiencing identical treatments of complete water withholding for four months (drought stress, DS) with no ground water in the basin or continuous well watering with maintaining the ground water level at -1.8 m (controls, CO), respectively. However, due to different morphological responses and properties, they in fact did not experience the same severity of drought stress (DS) during the experiment. After 4 months of withholding water, volumetric soil water content in the upper soil layers (10-20 cm depth) had decreased from 25-30 to below 10% in the DS basin. This resulted in significant decreases of predawn water potentials to -2.18 MPa (*Q. robur*, control [CO]: -0.38 MPa), -0.57 MPa (*Q. pubescens*, CO: -0.14 MPa) and -0.81 MPa (*Q. ilex*, CO: -0.13 MPa, as described in Früchtenicht *et al.* 2018b). Thus, while *Q. robur* (with the shallowest root system of the three species) was severely drought stressed, the other two species (with deeper rooting) experienced similar levels of moderate drought stress. This was also reflected in physiological parameters of the leaves: while the so-called performance index (PIabs) of the photosynthetic apparatus was significantly lower in DS *Q. robur* than in well-watered control trees; this was not the case in *Q. pubescens* and *Q. ilex*. PIabs is a chlorophyll fluorescence parameter summarizing various efficiencies of light collection and electron transport steps in the linear electron transport chain, which can be used as an indicator for *e.g.*, DS (Früchtenicht *et al.* 2018a).

### RNA sequencing output and pre-processing

A total of 961 million raw reads were sequenced (258 mill. for *Q. robur*, 316 mill. for *Q. pubescens*, and 386 mill. raw reads for *Q. ilex*). The PhiX contamination was on average 0.28%. Due to high quality of the raw reads, on average 97.9% were retained after pre-processing, resulting in 256 mill. reads for *Q. robur*, 313 mill. reads for *Q. pubescens*, and 383 mill. reads for *Q. ilex*.

### De novo assembly

After *de novo* assembly and further filtering of transcripts with less than five reads mapped, a total number of 335,571 transcripts for *Q. robur*, 420,306 for *Q. pubescens* and 100,158 transcripts for *Q. ilex* could be obtained. Best hit annotation against the whole UniProt Swiss-Prot databases showed that around 1% of the annotated transcripts were mouse (*Mus musculus*) contaminants in the *Q. ilex* assembly. Aligning the raw reads to the mouse coding sequences showed that the sample 23491 (see Table S1) from the *Q. ilex* control group contained a high number (3.64%) of mouse reads. To reduce false positive differentially expressed genes, transcripts that have more than 95% identical matches and an e-value ≤ 1e-5 with a mouse gene and/or protein were removed for further analysis. One explanation could be, that the experiment was performed in the Scientific Garden of Goethe University with significant populations of various wild-living animals, and that colonization of the experimental setup with mice was observed. Especially the dense leaf cover of *Q. ilex* may have attracted the animals as a shelter during their activities and may have given rise to contamination of the leaves.

After filtering of low expressed genes (by keeping genes with a CPM > 1 in at least two samples), which is done to increase the sensitivity of the analysis of differential gene expression (cf Sha *et al.* 2015), the assemblies consisted of 68,377 transcripts for *Q. robur*, 70,721 for *Q. pubescens*, and 53,111 transcripts for *Q. ilex*, with a N50 value of 911 bp, 768 bp, and 1,069 bp, respectively (Table 1).

**Table 1:** *De novo* assembly statistics for *Q. robur*, *Q. pubescens* and *Q. ilex*

	Transcripts	Transcripts after filtering	Genes	GC	N50	<150bp	Median	Mean
***Q. robur***	335,571	68,377	24,287	42.26%	911 bp	2%	519 bp	670 bp
***Q. pubescens***	420,306	70,721	27,983	42.23%	768 bp	2%	460 bp	583 bp
***Q. ilex***	100,158	53,111	26,221	41.18%	1,069 bp	0,40%	665 bp	825 bp

### Annotation

Using UniProt Swiss-Prot database more than 60% of all genes in the three *Quercus* species could be annotated with an e-value ≤1e-5 while even 84–88% of the genes could be annotated when using NCBI non-redundant database filtered for Viridiplantae sequences. A summary of the annotation is given in Table 2. In more than 90% of these annotations, the recently published *Quercus suber* genome (CorkOak1.0, GCF_002906115.1) is assigned as best hit, followed by *Juglans regia* (GCA_001411555.1) in 1.6% of all annotations (Table S2). GO terms could be assigned to more than 62% and Pfam protein domains to more than 47% of all genes from the three species. Transdecoder could predict proteins in more than 68%.

**Table 2:** Annotation statistics of assembled *Q. robur*, *Q. pubescens* and *Q. ilex* genes

	*Q. robur*		*Q. pubescens*		*Q. ilex*	
	Genes annotated	%	Genes annotated	%	Genes annotated	%
BLASTX (SP*)	16,192	67	17,821	64	16,560	63
BLASTP (SP*)	14,507	60	15,299	55	14,626	56
BLASTX (VP*)	21,395	88	24,198	86	22,121	84
BLASTP (VP*)	16,746	69	17,783	64	17,901	68
**GO**	15,969	66	17,600	63	16,165	62
**Pfam domains**	12,822	53	13,116	47	13,516	52
**Protein prediction**	17,754	73	18,943	68	18,320	70

* SP - UniProt Swiss-Prot database, *VP - NCBI non-redundant Viridiplantae database.

**Figure 1:**
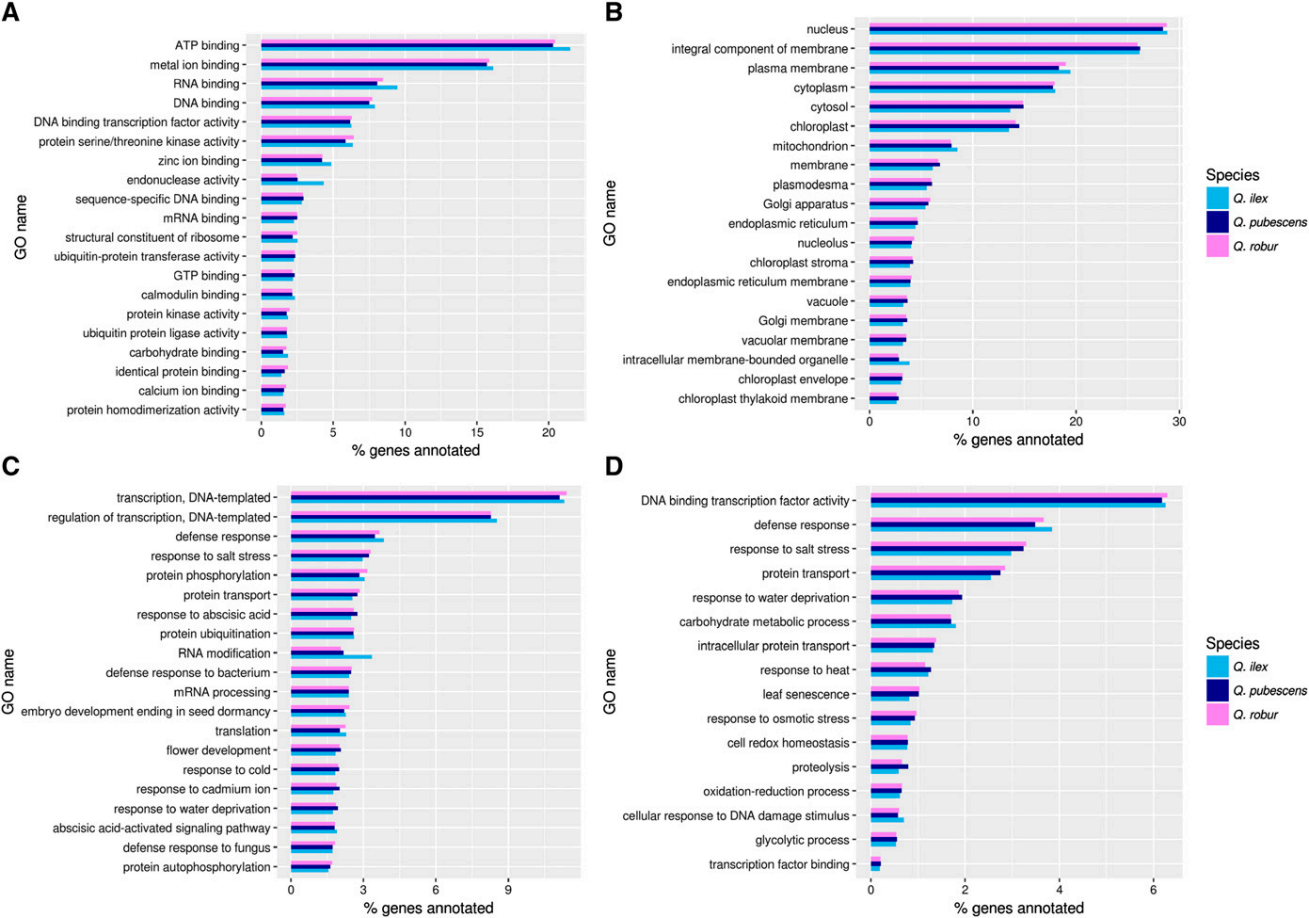
GO term distribution. 20 mostly assigned GO terms in the category (A) molecular function, (B) cellular function, (C) biological function and (D) selected drought stress related GO terms for *Q. robur* in comparison to *Q. pubescens* and *Q. ilex* in % of annotations relative to all GO annotated genes in that species.

**Figure 2:**
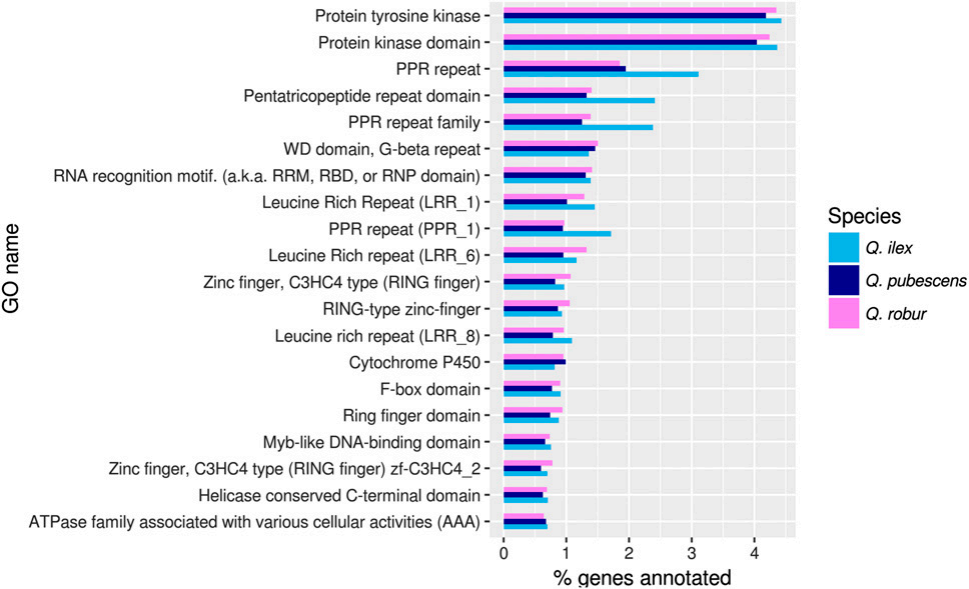
Pfam domain distribution. 20 mostly assigned Pfam domains for *Q. robur* in comparison to *Q. pubescens* and *Q. ilex* in % of annotations relative to all Pfam-annotated genes in that species.

The most frequent GO terms common to all three assembled leaf transcriptomes in the cellular component category are “nucleus”, “integral component of membrane” and “plasma membrane”; in the molecular function category “ATP binding”, “metal ion binding” and “RNA binding”; and in the biological process category “transcription, DNA-templated”, “regulation of transcription, DNA-templated” and “defense response” (Table S3). The GO terms “endonuclease activity” in the molecular function and “RNA modification” in the biological function category have an overrepresentation of genes in *Q. ilex* compared to the other two species (4.3% and 3.3% of genes, respectively *vs.* around 2.5% and 2%, respectively), see Figure 1A-D and Table S3. Pfam functional domain analysis showed that pentatricopeptide repeat domains are more frequently annotated for the *Q. ilex* assembly (Figure 2 and Table S4).

### Species-specific differential gene expression between CO and DS

**Figure 3:**
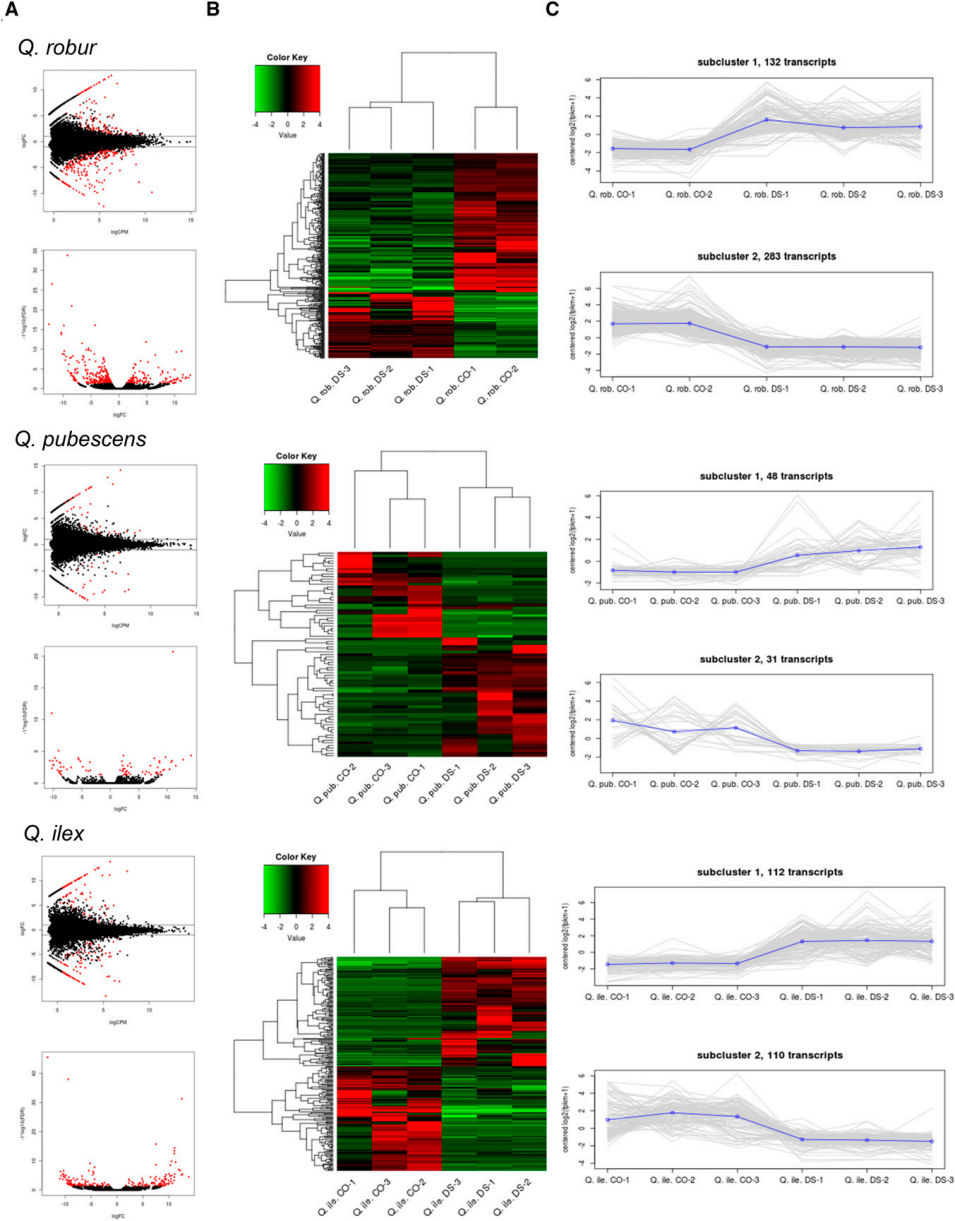
DEG analysis obtained using edgeR. MA plot in (A) shows log fold-change (logFC) *vs.* log average expression (logCPM). Significantly differentially expressed genes are shown in red. Volcano plot shows statistical significance (-log10 FDR) *vs.* log fold change (logFC). Hierarchical clustered heatmap in (B) showing significant differentially expressed gene abundance values (log2-transformed and mean centered FPKM values). Samples (control and drought stress) are clustered along the horizontal axis, differentially expressed genes along the vertical axis. Genes and samples are hierarchically clustered based on the Euclidean distance of gene abundance values and complete linkage clustering. Red and green color intensity indicate gene upregulation and downregulation, respectively. In (C) the expression patterns for each gene (grey) and the mean expression profile within that cluster (blue) is shown.

Considering 24,287 *Q. robur* genes, 415 genes were found significantly differentially expressed, with 132 higher and 283 lower expressed in the CO group than in the DS group. In *Q. pubescens*, where 27,983 genes were analyzed, 79 genes were found to be significant differentially expressed, with 48 genes significantly higher and 31 significantly lower expressed in the CO group compared to the DS group. The differential gene expression analysis of the 26,211 *Q. ilex* genes resulted in 222 significantly different expressed genes after water deprivation. 112 were significant higher and 110 were significant lower expressed in the CO group than in the DS group. A summary statistic for all species of the differential gene expression analysis and GO enrichment analysis is given in Table 3 and Figure 3, including MA and volcano plots, heatmaps and clusters.

**Table 3:** Summary of the species-specific differential gene expression and GO enrichment analysis

	Genes considered	DEGs	Sign. higher than CO	Not significant	Sign. lower than CO	BCV	Sign. enriched GO Terms
***Q. robur***	24,287	415	132	23,872	283	0.58	84
***Q. pubescens***	27,983	79	48	27,904	31	0.68	101
***Q. ilex***	26,221	222	112	25,999	110	0.66	35

**Figure 4:**
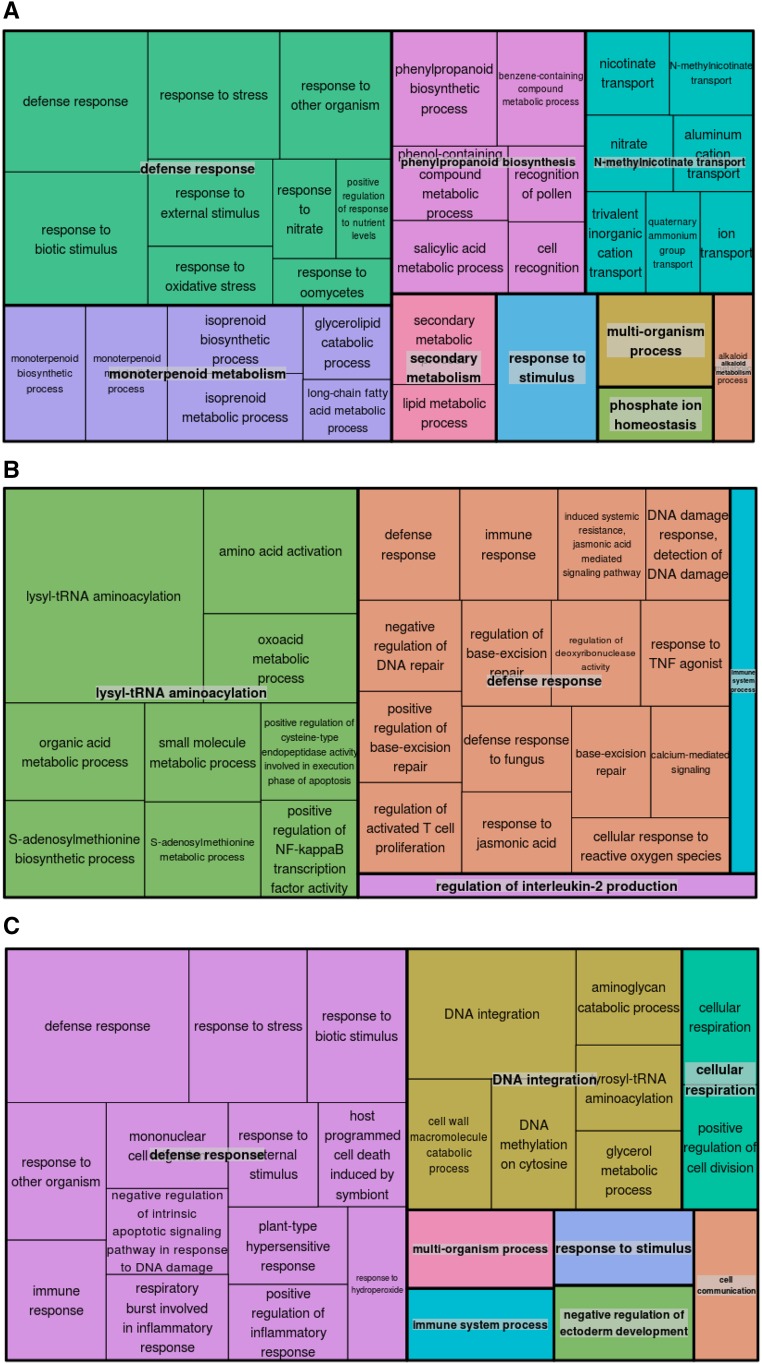
GO enrichment analysis for DEGs. Treemap containing most significant enriched GO terms (biological process) during drought stress, visualized with REVIGO for (A) *Q. robur*, (B) *Q. pubescens* and (C) *Q. ilex*, respectively.

GO enrichment analysis of the *Q. robur* differentially expressed gene dataset showed that 84 GO terms are significantly enriched. Most enriched GO terms in the category biological processes are “defense response”, “response to biotic stimulus” and “response to stress” (Table S5). Visualization (Figure 4A) of enriched GO terms shows further an enrichment of GO terms connected with phenylpropanoid and monoterpenoid metabolic processes (*e.g.*, ECOD) as well as ion transport (*e.g.*, YSL1 and OCT7) (Table S6).

The 40 top upregulated genes (FDR < 2.38E-03) include those encoding for the NRX2 and DOX1, proteins that protect the plant against oxidative stress. Further, a BURP domain protein RD22-like, a known dehydration-responsive protein, and a L-ascorbate oxidase-like was listed (Table S6). Further, we found the gene LEA known to play a major role in drought and other abiotic stresses tolerance in plants, and WAT1, an auxin transporter. Among the entire list of 132 upregulated genes, we additionally found two genes belonging to the NAC domain-containing proteins (*e.g.*, RD26), two related to sugar transport/metabolism (SPS4 and MSSP2). Among the 40 most downregulated genes (FDR < 4.54E-09), three cucumisin-like were listed, four genes that are annotated as LRR-RLKs (leucine-rich repeat receptor-like serine/threonine-protein kinases) and many other receptor-like proteins. Additionally, GLR2.8, the receptor-like protein EIX2, the beta-amyrin 28-oxidase, and two genes annotated as glutathione S-transferase U7-like were listed (Table S6). Further genes also found to be downregulated in the entire dataset of 283 genes were several sugar transporters (Table S6).

Besides a high enrichment of “defense response” GO terms in *Q. pubescens*, the GO enrichment analysis showed an enrichment of amino acid activation and especially (lysyl)-tRNA aminoacylation (Table S5 and Figure 4B). Concordantly to the GO enrichment analysis, five lysine-tRNA ligases were found significantly downregulated. Additionally, two genes annotated as F-box protein SKIP23, GLR3.3 and GLR3.6, the receptor-like protein EIX1, and cytochrome P450 87A3 were listed. Further downregulated were cellulose synthase-like protein E6, TERC, and linamarin synthase 2, which is involved in the biosynthesis of linamarin, a toxic cyanogenic glucoside (Table S7).

The significantly upregulated genes included genes involved in hormonal pathways, such as ERF4 and CYP74A1 / AOS1, involved in jasmonic acid biosynthesis, further two genes involved in sugar transport/metabolism (GPT2 and STP13), additionally, two genes of the phenylpropanoid pathway, PAL and 4CL, as well as PAT24 and the bHLH92 transcription factor, which is known to respond to osmotic stress. Further, two heat shock proteins were found, LOX3.1, BAT1, which is involved in GABA transport, and the E3 ubiquitin-protein ligase PUB23, that negatively regulates water stress response in *Arabidopsis thaliana* (Table S7).

In *Q. ilex*, the most enriched GO terms of the category biological processes were “defense response”, “DNA integration” and “response to stress” (Table S5). Notable are also an enrichment of “DNA methylation on cytosine” (CMT1 and DRD1) and processes involved in “cellular respiration” (XYLT) (Figure 4C and Table S8). In the list of the 40 most upregulated genes (FDR < 1.07E-03), the majority of genes is related to disease resistance including two that were annotated as LRK10, BDA1 and NPR4, both Ankyrin repeat-containing proteins that are regulators of plant immunity, and CDR1 and SUMM2. Further, three genes belong to the LRR-RLKs, additional three genes belong to SRKs, and COMT, involved in lignin biosynthesis, AOS3, involved in jasmonic acid biosynthesis, and OBE, genes that are required for the maintenance and/or establishment of both the shoot and root meristems, were included. Further, two genes homologous to cytochrome P450 genes: CYP75B1 and cytochrome P450 71A1. Considering the entire list of 112 genes, also a dehydration responsive protein, WAK5, MLP43 or EXPA1 were listed (Table S8).

Among many hypothetical and uncharacterized proteins in the 40 most downregulated genes (FDR < 4.66E-03), we found two genes annotated as CRK22, a putative disease resistance protein, and a LRR-RLK. Additionally, CUL1, a member of the SCF-complex, involved in jasmonate signaling, and MIK2, involved in cell wall integrity sensing, were found. Notable in the further list is also ZFWD1, DREB3, and two genes annotated as WAK1 and GAT1 (Table S8).

### Comparison of orthologous genes between species

To compare gene expression levels of genes that are present in all three species, orthologous groups of genes were identified across the species resulting in 14,005 orthologous groups that contain at least one gene of each species. As more genes of one species can be related to one orthogroup, in total 25,597 genes of *Q. robur*, 23,747 of *Q. pubescens*, and 22,616 of *Q. ilex* were assigned to orthogroups.

### Differences between oak species

The selection of the 200 orthologous genes with highest F-values (pairwise ANOVA test included in crossr workflow) of each comparison (*Q. robur - Q. pubescens*, *Q. robur - Q. ilex* and *Q. pubescens - Q. ilex*) led to 517 orthologous genes that show different expression in at least one species after DS (Table S9). Further visual examination of the expression patterns of these 517 orthologous genes could group 161 genes in categories that show a) a species-specific reaction, b) a different reaction pattern between deciduous and evergreen species, or c) a resemblance of the expression pattern to the gradually increasing DS tolerance of the three species, *Q. robur* < *Q. pubescens* < *Q. ilex*.

**Figure 5:**
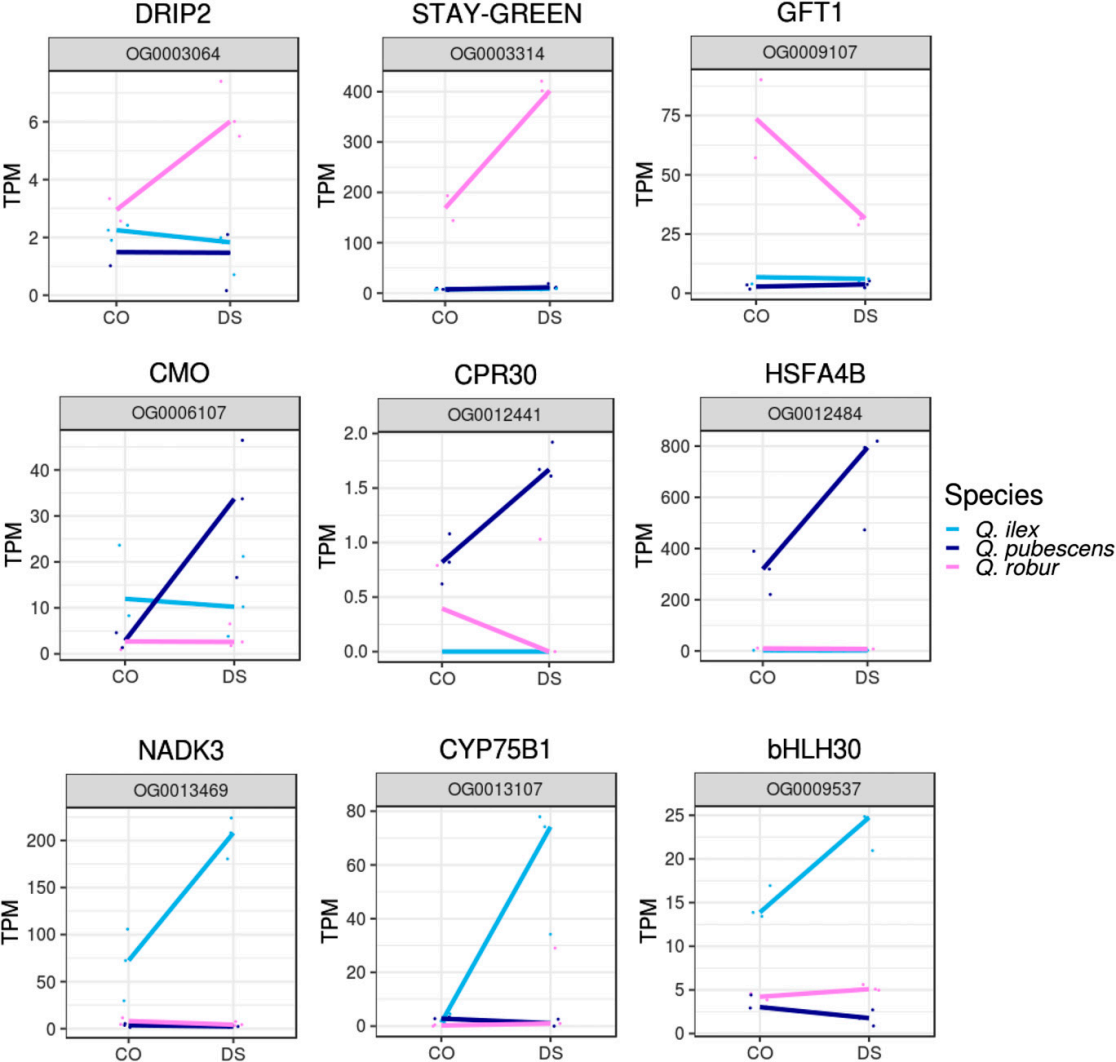
Species-specific response of orthologous genes during drought stress. Normalized expression values (TPM) of drought stressed (DS) and control (CO) samples of selected orthologous genes with species-specific drought stress response are shown for *Q. robur*, *Q. pubescens* and *Q. ilex*.
**b. Reaction pattern of deciduous compared to evergreen species**: Here, expression patterns of the orthologous genes are contrary for the deciduous species (*Q. robur* and *Q. pubescens*) compared to the evergreen species (*Q. ilex*) after DS. Among the 37 orthologous genes that might play an important role for the adaptive potential to DS in evergreen *Q. ilex* were EDR1, AOC, 4CLL7, FREE1, the GABA transporter 1 (GAT1), a 15.4 kD class V heat shock protein homolog (HSP15.4) and NADK3. The expression pattern of selected orthologous genes is shown in Figure 6.

**a. Species-specific reaction to drought stress:** Orthogroups that fall in this category only show a different expression pattern between CO and DS in one of the species while in the other two species an idle expression was seen. In total, 72 orthogroups were found, 56 specific for *Q. robur*, four for *Q. pubescens*, and 12 for *Q. ilex*. Selected orthogroups are depicted in Figure 5. Regarding *Q. robur*, 32 genes were up- and 24 were downregulated, among them, homologs of the E3 ubiquitin protein ligase DRIP2 and SGR, the first a master-regulator of DS tolerance and the latter involved in chlorophyll a degradation. Further, the sugar transporter GFT1. 14, MSSP2, WRKY53 and VEP1 were found in the species specific DEG analysis of *Q. robur*. DS led to the upregulation of four orthogroups specific for *Q. pubescens* genes, including choline monooxygenase (CMO) homolog, a homolog of the f-box protein CPR30, involved in the defense response, and a homolog of the heat stress transcription factor A-4a. *Quercus ilex* reacted to DS with the upregulation of 10 genes and with the downregulation of two genes, among them a NADK3 homolog, modulating abscisic acid responses, two genes homologous to cytochrome P450 genes: the flavonoid 3′-monooxygenase (CYP75B1), and a CYP71B5 homolog, the transcription factor bHLH30, and a homolog of a AMP deaminase.

**Figure 6:**
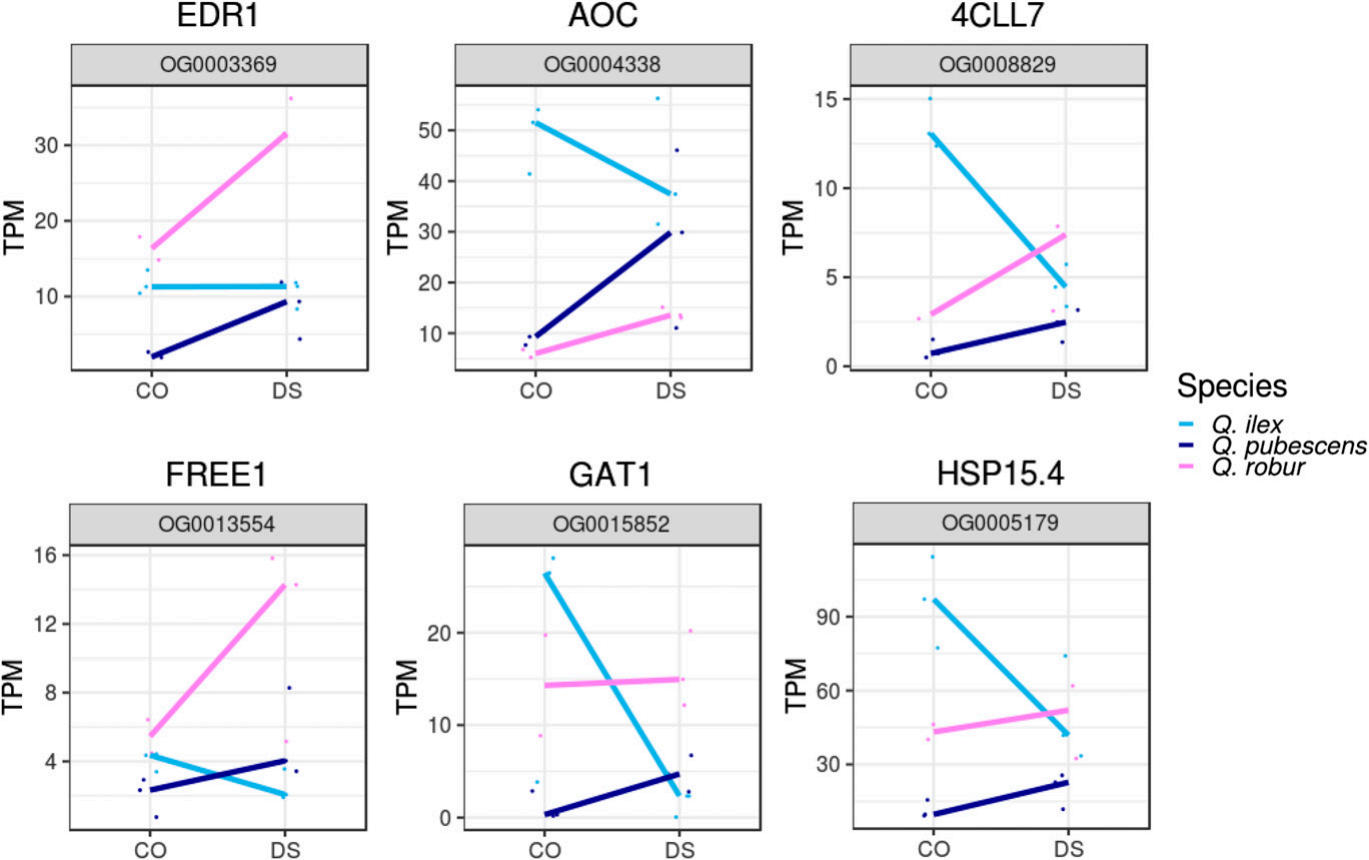
Different response of deciduous (*Q. robur*, *Q. pubescens*) and evergreen (*Q. ilex*) species during drought stress. Normalized expression values (TPM) of drought stressed (DS) and control (CO) samples are shown for *Q. robur*, *Q. pubescens and Q. ilex*.
**c. Response along the DS-tolerance gradient**: To encounter genes that are connected to the gradual DS tolerance which is present in the three species, we compared the not so DS tolerant *Q. robur* to the semi DS tolerant *Q. pubescens* with the DS tolerant *Q. ilex* to find patterns resembling this gradient as well as genes that follow the gradient of the measured level of water potential. The expression pattern of selected orthologous genes can be found in Figure 7. Among the 17 orthologous genes that show a gradually increasing gene expression with increasing DS tolerance, starting from a downregulation in *Q. robur*, a rather stable expression in *Q. pubescens*, and an upregulation in *Q. ilex*, a dnaJ protein ERDJ2-like homolog, a folylpolyglutamate synthase (FPGS), two homologs of TWINKLE, which is known to be involved in organelle DNA replication, a LRK10 homolog, and a 28 kD heat- and acid-stable phosphoprotein-like gene were observed. *Vice versa*, among the 11 genes that were characterized by an upregulation of gene expression in *Q. robur* and a downregulation in *Q. ilex* (with a stable expression in *Q. pubescens*), we found the indole-3-acetic acid-induced protein ARG7, the TCP9 TF, MTERF5, involved in ABA-related stress response, and a Zinc finger CCCH domain-containing protein 63 homolog. Furthermore, 21 genes showed downregulation in *Q. robur* and an upregulation in *Q. pubescens* (and a stable expression in *Q. ilex*), *e.g.*, AGO5, the guanine nucleotide-binding protein-like NSN1, homologs to VQ motif-containing protein 22 (VQ22, JAV1) and to the WRKY transcription factor 33 / WRKY24-like, whereby VQ proteins are known to interact with WRKY transcription factors (Chi *et al.* 2013), a homolog to heavy metal-associated isoprenylated plant protein, shown to be involved also in transcriptional responses to cold and drought. And further 13 genes showed an upregulation in both, *Q. pubescens* and *Q. ilex*, and a downregulation in *Q. robur*. Among them, the cinnamoyl-CoA reductase 1, involved in lignin biosynthesis, PAO1, ACOX1, involved in jasmonate biosynthesis, plant L-ascorbate oxidase (AO), a homolog of a known stress-responsive GST gene, and a scopoletin glucosyltransferase-like homolog (TOGT1).

**Figure 7:**
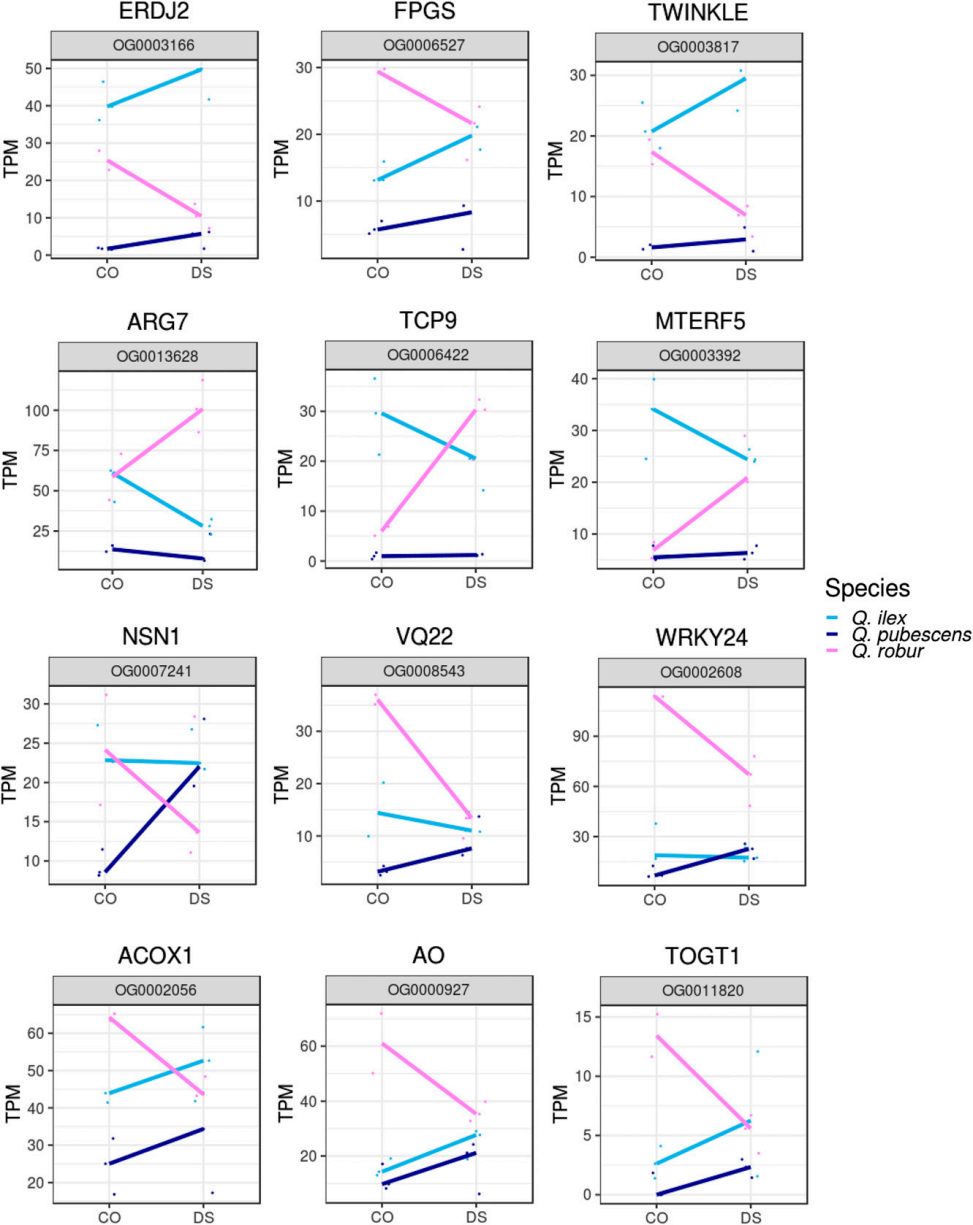
Gradually different response in *Quercus* species. Normalized expression values (TPM) of drought stressed (DS) and control (CO) samples of selected orthologous genes that behave gradually different in *Quercus* species during DS are shown.

### Reaction upon DS on the genus level

**Figure 8:**
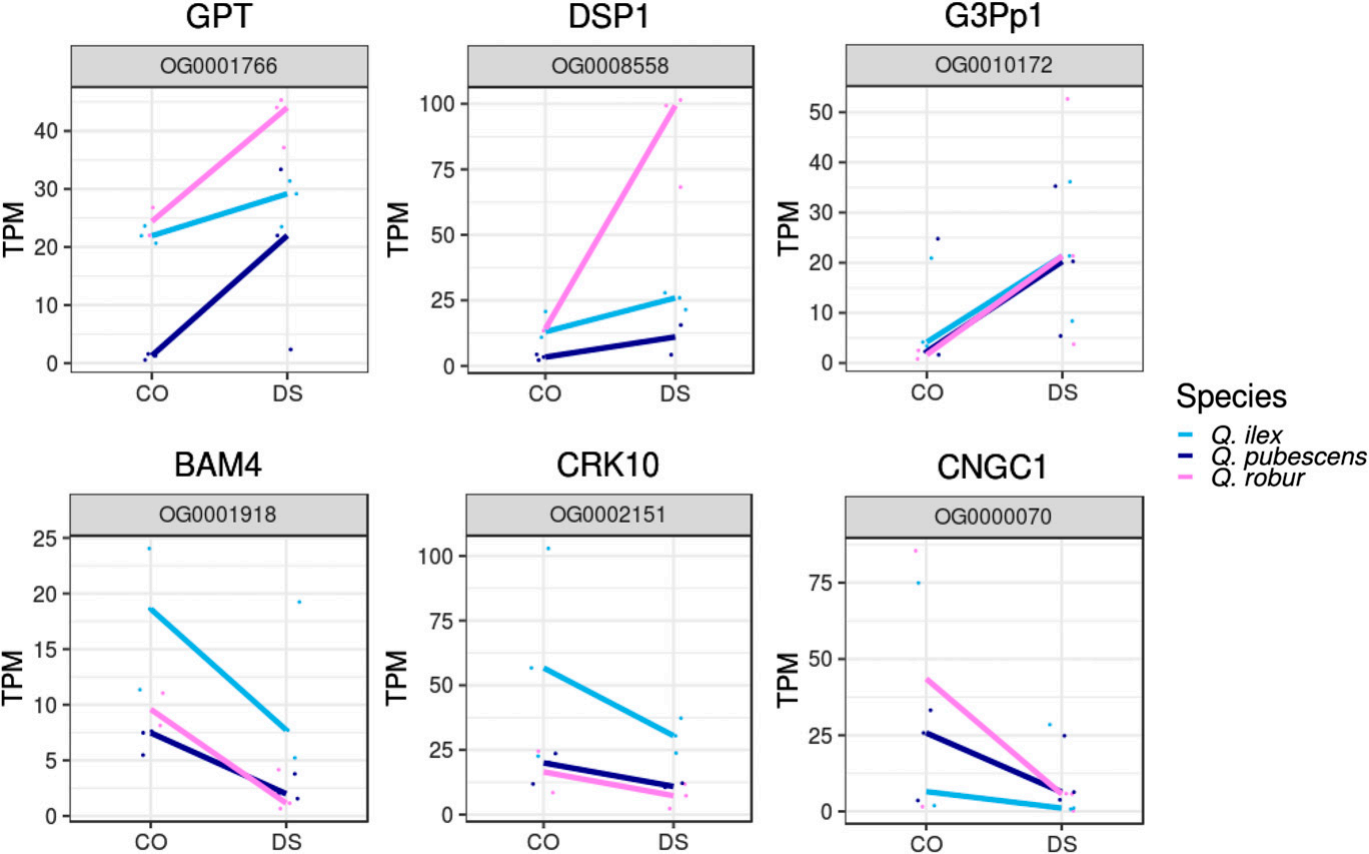
Orthologous genes with similarly response during drought stress in all *Quercus* species. Normalized expression values (TPM) of drought stressed (DS) and control (CO) samples are shown for selected orthologous genes that are similarly up or downregulated in all species.

Using the strict criteria for individual DEG analysis (FDR < 0.05 and |logFC| > 1), no orthologous groups were found that contain significantly differentially expressed genes for all species after DS. However, considering a p-value < 0.1, five orthologous groups were found to be upregulated, while nine were found to be downregulated similarly across all three *Quercus* species (Table S10). Among the upregulated genes, a homolog to the glucose-6-phosphate/phosphate-translocator (GPT), DSP1 and G3Pp1 were listed (Figure 8). Among the downregulated orthologous genes, CRK10, CNGC1, and BAM4 were found (Figure 8).

## Discussion

This study describes the species-specific DS response on the transcriptional level of three European oak species with respect to various biological processes, such as hormonal pathways, osmoprotection, photosynthesis and cell wall remodeling. Through the comparison of orthologous genes present in all three species, we were able to point toward differences between deciduous and evergreen species as well as highlight genes whose transcriptional behavior correlates to the DS-tolerance gradient, from the least to the most tolerant species.

When drought occurs during vegetative growth, plants react in a flexible way and reprogram growth (for reviews see Claeys and Inzé 2013; Pierik and Testerink 2014). In this respect, an important feature is a drought avoidance strategy including the rearrangement of root architecture and increasing the root/shoot ratios, thus ameliorating the negative effect of a naturally drying soil on plant water supply (for reviews Malamy 2005; Brunner *et al.* 2015). Root/shoot ratios also depend on latitudinal origin and season, more northern provenances show a higher root/shoot ratio at the end of the growing season (Cannell and Willett 1976). In the current study, the root depth was measured in autumn: *Q. pubescens* showed the deepest root system (-165 cm), followed by *Q. ilex* (-150 cm) and *Q. robur* (-100 cm) (Früchtenicht *et al.* 2018b); results that correlate with the different severity of the DS experienced by the leaves, but not with origin or genotype of the plant material. Under the severe stress experienced by the *Q. robur* leaves, stomatal closure over long periods is unavoidable, resulting in CO_2_ deprivation and Mehler reaction with subsequent ROS stress. According to the Ψ_PD_ values above -0.8 MPa, *Q. pubescens* only experienced very mild DS (Früchtenicht *et al.* 2018b), nevertheless, we found a decent response to DS on the transcriptional level.

### Species-specific response to DS

As a general feature, *Q. robur* revealed much more changes in gene expression (415 differentially expressed genes between CO and DS) than the two Mediterranean (and more drought tolerant) species, in accordance with its much lower water potential during water withholding despite the identical abiotic conditions, to which the three species were exposed. Apparently, while *Q. pubescens* and *Q. ilex* were in a state of drought avoidance/drought resistance reactions, *Q. robur* already suffered severely from the treatment, resulting in more fundamental changes in metabolism and the onset of catabolic processes.

#### Q. robur:

As expected, the analysis of GO terms (“biological processes”) showed a significant enrichment of “defense response”-related GO terms (biotic and abiotic) in all three species, however, *Q. robur* further showed an enrichment of GO terms related to “secondary metabolism” (*e.g.*, phenylpropanoid, monoterpenoid), an indication that the antioxidant machinery was induced. This is also supported by the expression of ROS scavengers, NRX2 and DOX1, known to reduce oxidation (De León *et al.* 2002; Kneeshaw *et al.* 2017). Also, sucrose was produced (SPS4), likely functioning as ROS scavenger, too (Solís-Guzmán *et al.* 2017). Cell integrity/stability and cell wall composition both play important roles in DS response and tolerance mechanisms (Tenhaken 2014; Feng *et al.* 2016). Through the activation of ABA/auxin-mediated gene expression (RD22, WAT1) and further genes related to cell wall remodeling (AUG8, PDCB3), *Q. robur* most likely induced its adaptation to drought by strengthening the secondary cell wall of fibers (Yamaguchi-Shinozaki and Shinozaki 1993; Ranocha *et al.* 2013; Shi *et al.* 2014; Phillips and Ludidi 2017) and by closing plasmodesmata (Sager and Lee 2014). Furthermore, under DS, the photosynthetic apparatus is extremely stressed due to stomatal closure and subsequent CO_2_ depletion (Früchtenicht *et al.* 2018a). In our dataset, DRIP2 was upregulated suggesting an inactivation of DREB2A-mediated gene expression in the long-term response to DS in *Q. robur* (Qin *et al.* 2008), which further hints toward a higher susceptibility to DS (Sakuma *et al.* 2006) in this species in comparison to *Q. pubescens* and *Q. ilex*. Further, catabolism in form of chlorophyll a breakdown (SGR) was induced (Sakuraba *et al.* 2014; Sato *et al.* 2018), supporting previous studies, where significantly lowered chlorophyll content during DS were reported (Früchtenicht *et al.* 2018a). But, repair mechanisms (DEGP1) of PSII damages already started (Sun *et al.* 2010; Kley *et al.* 2011; Zienkiewicz *et al.* 2013). Effects of DS on genes involved in carbohydrate metabolism were found in all three oak species. Sugars are not only the product of photosynthesis, and thus subject to drastic concentration changes under DS, but may also play a role as osmotica in oaks (Épron and Dreyer 1996). In *Q. robur*, sugar synthases and transporters were activated (SPS4, MSSP2, GPT2) but also downregulated (GFT1, SWEET7), likely contributing to osmotic adjustment to maintain metabolic activity in source leaves (Büttner and Sauer 2000; Rautengarten *et al.* 2016; Solís-Guzmán *et al.* 2017). Regarding control mechanisms on the molecular level, many transcription factors (TFs) are described to be drought-inducible. Confirming earlier results on NAC TF activation after drought in *Q. robur* (Spieß *et al.* 2012), NAC domain-containing proteins were found to be upregulated also in our study after DS, likely leading to drought-induced senescence, fiber development and further abiotic stress responses (Podzimska-Sroka *et al.* 2015; Wu *et al.* 2016). Interestingly, both members of the DET1-HY5 pathway were activated, suggesting DS-induced leaf curling in *Q. robur* (cf Abrams 1990). A well-known family of proteins related to heat stress and other abiotic stresses are heat shock proteins (HSPs) (De Maio 1999), among them one group called the Late embryogenesis abundant (LEA) proteins. In this context, Spieß *et al.* (2012) already described the upregulation of a LEA by DS in *Q. robur*, which could be confirmed in our study, indicating that this species increased its tolerance to dehydration through chaperone activity (Hong-Bo *et al.* 2005).

#### Q. pubescens:

Interestingly, in *Q. pubescens* much less genes (n = 79) were significantly differentially expressed after DS in comparison to *Q. robur* (415) and *Q. ilex* (222). The reason could be that the predawn water potential (see Table 1 in Kotrade *et al.* 2019) showed the less negative water potential (-0.47 ΨPD) and the least difference in water potential between CO and DS trees on the DOYs of sampling (287 and 288), indicating that the DS perceived on leaf cellular level was lower in *Q. pubescens* as compared to *Q. robur* and *Q. ilex* in the chosen, competitive experimental setup. Concerning its water potential, *Q. pubescens* only reached a level of mild DS. Nevertheless, it reacted to water withholding with the induction of jasmonic acid (JA) biosynthesis through LOX3.1 and AOS1, a process, that might play also an important role in the formation of antioxidants (Hause *et al.* 2002). Besides JA, also GABA plays an important role in DS tolerance (Shelp *et al.* 1999; Li *et al.* 2016, 2017; Mekonnen *et al.* 2016). The upregulation of the GABA permease BAT1 in *Q. pubescens* could hint toward the onset of catabolic processes by reducing the protein synthesis machinery, activating protein degradation, and subsequently export of amino acids from leaves (Dündar and Bush 2009). This can be supported by the GO analysis, that detected an enrichment of amino acid activation and especially (lysyl)-tRNA aminoacylation after DS treatment, resulting from significant downregulation of genes annotated for lysine–tRNA ligase, *e.g.*, lysyl-tRNA synthetase (KRS) (Wu *et al.* 2007). The important role of ROS scavenging, sugar allocation and regulation of osmotic state in *Q. pubescens* after the drought treatment was further reflected in the differential expression of four genes related to these processes: GPT2, STP13, CMO, and bHLH92 (Yamada *et al.* 2011). Further, enhanced root growth could mediate DS tolerance in *Q. pubescens* by the upregulation of the F-box protein CPR30, a gene that may interact with multiple ASK proteins, which are known to be involved in regulating stomatal opening and root growth (Li *et al.* 2012). Like *Q. robur*, *Q. pubescens* activated heat shock proteins (ERD8/HSP80/HSP90.2, HSP83/HSP90.1) and heat stress transcription factors (HSFA4b) to maintain correct folding and protection of protein degradation (Clément *et al.* 2011; Pérez-Salamó *et al.* 2014). A possible adaptational mechanism to drought stress in *Q. pubescens* could be the strengthening of the cell wall through the incorporation of lignin by the activity of PAL, 4CL, and COMT (Moura *et al.* 2010; Pandey *et al.* 2010; Byeon *et al.* 2014; Le Gall *et al.* 2015; Wang *et al.* 2017), while the depletion of thylakoid membranes in chloroplasts through TERC may lead to a decrease in photosynthesis, most likely a starting point of catabolic processes (drought-induced senescence) (Batra *et al.* 2014; Schneider *et al.* 2014).

#### Q. ilex:

Next to a significant enrichment of “defense response”-related GO terms (biotic and abiotic), *Q. ilex s*howed additionally an enrichment in GO terms of “cytosine methylation” and “cellular respiration”. Interestingly, in evergreen cork oak (*Q. suber*), an increase of cytosine methylation has been reported in heat-treated leaves (Correia *et al.* 2013), suggesting that *Q.ilex* may use the same mechanism of DNA structure reorganization to deal with DS. *Quercus ilex* reacted upon drought similarly as *Q. pubescens* by the activation of JA biosynthesis and signaling through AOS3 and CUL1 (Hause *et al.* 2002; Nagels Durand *et al.* 2016) and by increasing ABA-mediated drought tolerance (MLP43; Wang *et al.* 2016), most likely through ABA-responsive gene expression (bHLH30 with *Arabidopsis* homolog bHLH32; 94). As in the other two species, oxidative stress and the production of ROS is scavenged, in this case through the production of NADP(H) via the activity of NADK3 (Mittler 2002; Li *et al.* 2018) and through the production of flavonoids via CYP75B1 (Narusaka *et al.* 2004; Fini *et al.* 2011). It is likely that DS also impaired the flowering time through deactivation of COL14, which is supported by a study showing a 30% decrease in flower production of *Q. ilex* by drought (Ogaya and Peñuelas 2007). One of the major responses to DS in *Q. ilex* involved genes related to cell wall remodeling conferring drought tolerance, like COMT, CSLE6, MIK2, ARAD1, WAK1, WAK5 and ExPA1 (Harholt *et al.* 2006; Moura *et al.* 2010; Kohorn and Kohorn 2012; Le Gall *et al.* 2015; Feng *et al.* 2016; Van der Does *et al.* 2017). Further, many LRR-RLKs, SRKs and CRKs responded to DS (*e.g.*, LRK10), indicating their important regulatory function in this species (Marshall *et al.* 2012; Lim *et al.* 2015; Lu *et al.* 2016). Interestingly, the Pfam functional domain analysis showed that PPR (pentatricopeptide repeat) domains are more frequently annotated for the *Q. ilex* assembly than for the other two species (Figure 2). PPR proteins are involved in all aspects of organelle RNA metabolism (Yagi *et al.* 2014) and a recent study (Xing *et al.* 2018) showed that 154 PPRs in *Populus* were induced by biotic and abiotic treatment. However, roles of PPR genes in higher plant species remain largely unknown (Xing *et al.* 2018).

### Differential response between deciduous and evergreen species

Through the comparison of orthologous genes, we found 37 that show a contrasting expressional behavior between the deciduous (*Q. robur*, *Q. pubescens*) and the evergreen species (*Q. ilex*) after DS perception. In deciduous oaks, GAT1 (GABA transporter 1) was activated but repressed in the evergreen species, indicating that the turnover and likely the accumulation of GABA is one of the mechanisms for adaptation to drought in the deciduous species. Also, JA (jasmonic acid) and ethylene seemed to be synthesized preferably in deciduous oaks through the activation of AOC, 4CLL7, and ERD1, but repressed in evergreens. These genes were already shown to be related to drought but could also be further associated with leaf senescence (He *et al.* 2002; Tang *et al.* 2005; Moura *et al.* 2010; Li *et al.* 2017), a process, that might have been already initiated in the deciduous species after DS. In addition, the small heat shock protein HSP15.4 and S1P were activated in DS deciduous but repressed in DS evergreens, indicating that deciduous oaks prevented DS damage by increased chaperone activity (Al-Whaibi 2011) and by stimulating the endoplasmic reticulum (ER) stress signaling pathway, which can occur when unfolded or misfolded proteins accumulate in the ER (Liu *et al.* 2007). Vacuolar protein transport seemed to be enhanced in deciduous species through the upregulation of FREE1 (Gao *et al.* 2015). Furthermore, out of the 37 orthologous genes, 12 showed a differential pattern only in the evergreen species between the CO and DS cohort, among them, orthologous genes that are involved in ROS scavenging (NADK3, CYP71B1 and CYP71B5) as well as bHLH30, that might confer ABA-mediated drought tolerance (Kim and Kim 2006), and genes that have been discussed before in the species-specific response pattern.

### Response along the DS-tolerance gradient

Comparing *Q. robur* with the more drought tolerant *Q. pubescens* and *Q. ilex*, we found a higher number of genes involved in ROS scavenging in the DS cohorts of the more drought tolerant species. Maintaining a certain ROS level involves several proteins that are either involved in ROS production or ROS scavenging. FPGS2 for example, a regulator of cellular folate homeostasis (antioxidant activity), is upregulated in *Q. ilex* but downregulated in *Q. robur*. Similarly, increased carotenoid and ascorbic acid (AsA) contents improve tolerance to abiotic stresses through their antioxidant activity (Akram *et al.* 2017), exemplified in the expression pattern of PDS (downregulated in *Q. robur* and upregulated in *Q. ilex*), a gene that is involved in carotenoid biosynthesis (Hugueney *et al.* 1992). L-ascorbate oxidase (AO) upregulation in the Mediterranean species and downregulation in *Q. robur* possibly is connected to the latter’s lower DS tolerance (Fotopoulos *et al.* 2008). ROS scavenging as a predominant DS tolerance process is also reflected in the upregulation of GST and scopoletin glucosyltransferase (TOGT1) in *Q. pubescens* and *Q. ilex*, which are involved in ROS detoxification by glutathione (Xu *et al.* 2015) and scopolin (Zandalinas *et al.* 2016; Döll *et al.* 2018), respectively. Besides the reduction of ROS, increased DS-tolerance is possibly also mediated by the maintenance of the mitochondrial respiration machinery (Atkin and Macherel 2009), as can be seen through the increased expression of TWINKLE in *Q. pubescens* and *Q. ilex*, a gene involved in mitochondrial DNA replication (Diray-Arce *et al.* 2013) and by the repression of mTERF5, a gene involved in the regulation of mitochondrial gene expression (Jõers *et al.* 2013). Further, these species activated the biosynthesis of lignin through the expression of CCR1 (Srivastava *et al.* 2015), required for lignification of interfascicular fibers and the xylem (Vanholme *et al.* 2010). The inactivation of PAO1, observed in *Q. robur*, together with PAO5, has already been proven to confer tolerance to drought in *A. thaliana* (Sagor *et al.* 2016).

Regarding the hormonal cross-talk in response to DS, most of the genes that were upregulated in *Q. robur* but downregulated in the tolerant species were linked to hormonal pathways, *e.g.*, TCP9 transcription factor, involved in the control of leaf development via the jasmonate signaling pathway (Danisman *et al.* 2012), the indole-3-acetic acid-induced protein ARG7 and the RNA-binding protein ZFWD2, indicating that in the severely stressed *Q. robur* further phytohormone-related DS responses were activated. The DS tolerant species also activated the heat shock protein machinery. ACX1, a gene that is described to be induced by DS was upregulated in *Q. pubescens* and *Q. ilex*. It interacts directly with HSP90, one of the key chaperones involved in modulating a multitude of cellular processes under both physiological and stress conditions (Chong *et al.* 2015). ERDJ, which is a drought inducible co-chaperone component of the HSP70 system (Ohta and Takaiwa 2014), was upregulated in *Q. ilex*. The overexpression of a homologous gene in *Nicotiana*, NtDnaJ1, enhanced drought tolerance possibly through regulating expression of stress-responsive genes (Xia *et al.* 2014). The combined upregulation of VQ22 and WRKY33 in DS *Q. pubescens* is connected to the regulation of downstream gene expression. This transcriptional regulation could confer drought (and heat) tolerance in deciduous oaks since VQ22-WRKY33 binds to the promoter region of stress-responsive genes (Li *et al.* 2011; Jing and Lin 2015). Another putative drought tolerance-inducing gene that was downregulated in *Q. robur* only is NSN1, which leads to growth retardation and premature senescence when silenced (Jeon *et al.* 2015).

Taken together, antioxidant activity seems to be one of the major characteristics for enhanced DS tolerance in oaks. In the more tolerant species, many genes involved in the production of antioxidants (vitamins, carotenoids) and in ROS scavenging were upregulated compared to the less tolerant species. Also, the maintenance of the mitochondrial respiration machinery, the lignification of the water transport system and the suppression of drought-induced senescence might confer a higher tolerance to DS in *Q. pubescens* and *Q. ilex* compared to *Q. robur*.

### Common response to drought on the genus level

Despite the fact, that Old World oaks (Group Ilex, incl. *Q. ilex*) were separated from the New World oaks (Group Quercus, incl. *Q. robur* and *Q. pubescens*) more than 50 million years ago in the early Eocene (Hubert *et al.* 2014), highlighting a large difference on sequence level between the two groups, and an estimated divergence of the European white oaks 5 million years ago (Hubert *et al.* 2014; Leroy *et al.* 2017), we found 14 orthologous groups, that showed the same expression pattern across the three *Quercus* species, among them the gene CRK10 of the CRR-RLK family, which was downregulated in response to DS in all three species. The role of RLKs in responses to DS has been discussed already for plants in general (Marshall *et al.* 2012) and for *Q. ilex* in this study (see above). As suggested by the diversity and size of the RLK family (with more than 600 and 1,100 family members in *A. thaliana* and rice, respectively; Shiu *et al.* 2004), a genus- or even a species-specific concerted response pattern of its members to DS can be expected. Tolerance to DS on the genus level might additionally be mediated by the downregulation of BAM4, that is involved in starch breakdown (Kossmann and Lloyd 2000), and its activity is known to be induced by several abiotic stresses (Kaplan and Guy 2004). A beta-amylase 1 mutant in *A. thaliana* exhibited improved tolerance to DS through reduced starch breakdown in guard cells which led to decreased stomatal opening (Prasch *et al.* 2015). In many plant species, sugar transport to the cytoplasm plays a role in increasing DS tolerance (Yamada and Osakabe 2017). In *Q. petraea*, total soluble carbohydrates (sucrose, glucose, fructose) increased upon water shortage, thereby contributing to drought-induced osmoregulation (Épron and Dreyer 1996). In our analyzed oak species, we found an upregulation of GPT, a gene that is involved in the transport of glucose 6-phosphate across plastid membranes, and G3Pp1, which is involved in the glycerol-3-phosphate shuttle. G3P is a well-known inducer of systemic immunity in plants (Chanda *et al.* 2011) and our results indicate its involvement in drought stress perception. Apparently, oaks respond to DS by activating hexose allocation to the cytosol for osmotic adjustment. In addition, the upregulation of DSP1 indicates an increased sensitivity toward drought stress in all three species (Liu *et al.* 2015). Interestingly, through the downregulation of CNGC1 after DS perception, one could speculate, that stress-induced calcium uptake into oaks is hindered and as an effect, root growth might be enhanced (Ma *et al.* 2006; Jha *et al.* 2016; Wilkins *et al.* 2016) likely with the attempt to reach a water-bearing soil level.

## Conclusion

A plant’s answer to DS is manifold and depends on many environmental factors as well as on species-specific or even individual-specific mechanisms and preconditions. The analysis of orthologous genes that reacted the same in response to DS on the genus level revealed the induction of commonly known drought-responsive transcription factors and kinases, as well as genes involved in cell wall remodeling and lignification. Our data further indicate, that DS tolerance on the genus level could be mediated by a reduced starch breakdown, and further by an elevated sugar and glycerol-3-phosphate transport. At the species level, *Q. robur* reacted to the severe stress reflected in the very low water potential by activating ABA-mediated gene expression, the ROS scavenging machinery, and adjusting the cellular osmotic potential to maintain metabolic activity in the cell. However, catabolic reactions (chlorophyll breakdown) started, possibly indicating the onset of drought-induced senescence. Despite the late sampling date, the catabolic reactions are probably not caused by daylength-induced senescence processes since they were not detected in the CO group and the sampling date was chosen before major chlorophyll breakdown starts in *Q. robur* according to a previous study (Holland *et al.* 2014). Although *Q. pubescens* experienced a much lower level of DS on the cellular level compared to *Q. robur* (see Früchtenicht *et al.* 2018b), similar molecular mechanisms were activated, such as ROS scavenging for cell detoxification, and genes involved in root growth and the regulation of stomatal opening, but also catabolic processes leading to a decrease in photosynthesis. Differently than *Q. robur*, *Q. pubescens* activated the heat‐shock protein/chaperone network as well as lignification. In the most tolerant species, *Q. ilex*, also the activation of the ABA-mediated response to DS (as *Q. robur*) was detected, but in addition predominantly genes related to ROS scavenging and cell wall remodeling were regulated, which might help allowing to maintain growth and vigor after DS perception.

Taken together, a higher antioxidant activity seems to be one of the major characteristics for enhanced DS tolerance in oaks. But also the maintenance of the mitochondrial respiration machinery, the lignification of the water transport system and the suppression of drought-induced senescence might confer a higher tolerance to DS; a valuable knowledgebase of putative candidate genes, that could be integrated in breeding strategies for drought tolerant oaks.
